# Lausanne medical dispatch centre’s response to COVID-19

**DOI:** 10.1186/s13049-020-00735-8

**Published:** 2020-05-13

**Authors:** Fabrice Dami, Vincent Berthoz

**Affiliations:** 1 Emergency Department, Lausanne University Hospital, University of Lausanne, CH-1011 Lausanne, Switzerland; 2 Fondation Urgences-Santé, Lausanne medical dispatch, Lausanne, Switzerland

**Keywords:** Dispatch, COVID-19, Help-lines

## Abstract

The COVID-19 crisis is an unprecedented event. It is therefore essential for dispatch centres to share their experiences while the crisis is underway, similar to hospitals, so that we will all benefit from feedback.

This letter to the editor describes the Lausanne dispatch centre response to COVID-19 and the lessons learned so far.

## Main text

Sir,

The COVID-19 crisis is an unprecedented event. It is therefore essential for dispatch centres to share their experiences while the crisis is underway, similar to hospitals, so that we will all benefit from feedback [1].

Lausanne dispatch only maintains medical response services and is staffed by registered nurses and certified paramedics with at least 5 years of field experience. It is a criteria-based system that covers a population of 1 million inhabitants. The dispatch handles only high acuity calls requiring an ambulance or a helicopter, fielding approximately 300 calls and 150 missions per day. A second non-emergency medical help-line dispatch is located in the same building, handles 800 calls per day and is staffed with registered nurses only. This dispatch handles low acuity calls and offers counselling; when appropriate, a general practitioner will be sent to the patient’s home. On a daily basis, this second dispatch protects the emergency dispatch from low acuity calls or requests of information, so emergency dispatchers remain available for high acuity calls.

Medical dispatch is the first link of the chain of survival. Its primary goal is to remain reachable and functional at all times to provide the necessary assistance and it must answer all calls ideally within 10 s.

On February 20th, the first COVID-19 case was confirmed in Switzerland. On March 5th, we registered the first death due to the COVID-19 in our region. Before the pandemic reached hospitals, it started many days prior with dispatch response.

**Fig. 1:**
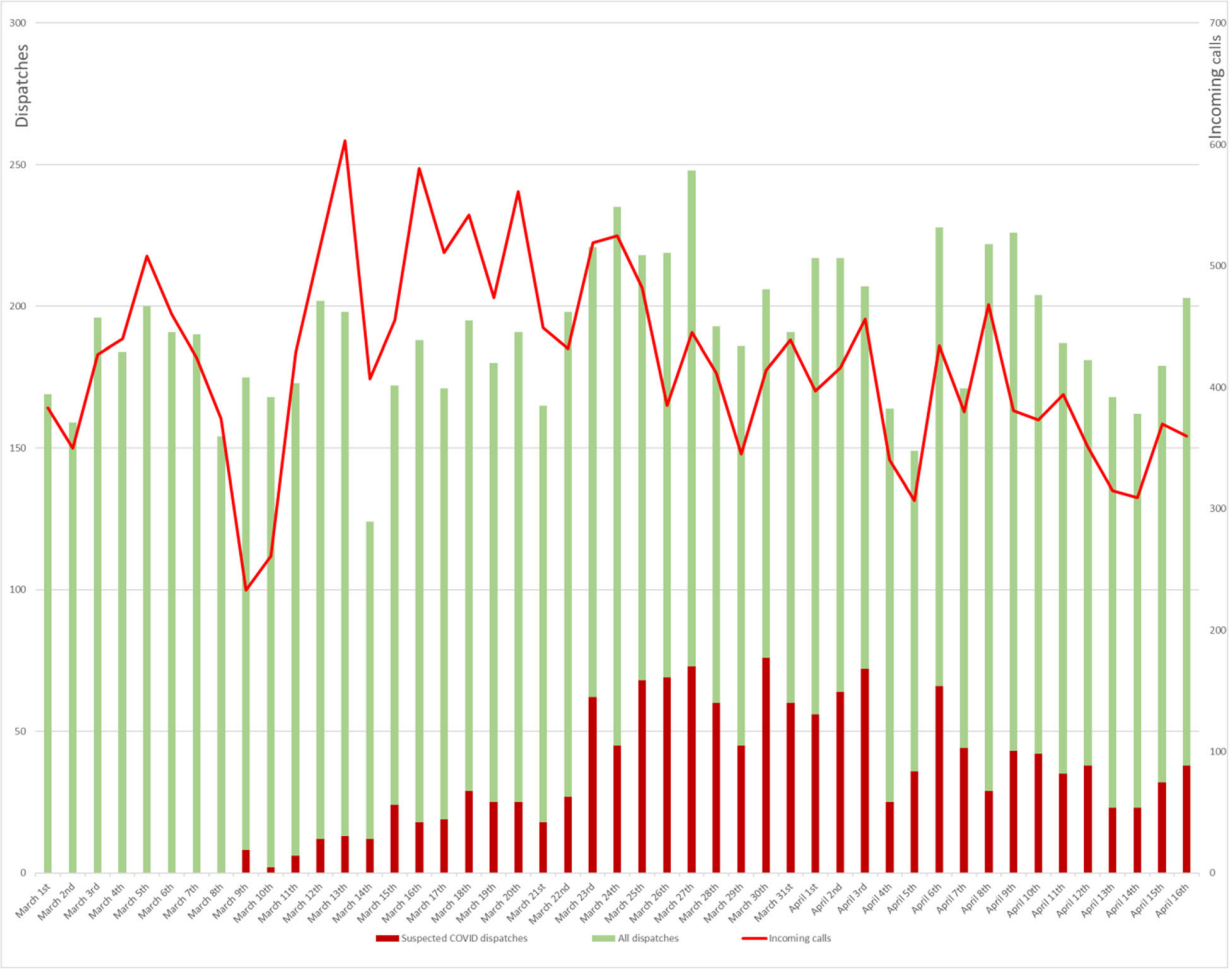
Volume of incoming calls and dispatches (COVID-19 and total) from March 1st to April 16th 2020

The first risk dispatch centres face during any major incident is to become overloaded with calls, whether these are true emergencies or not, and lose the ability to respond to requests for urgent relief. Figure 1 shows the number of calls received and ambulances dispatched (total and COVID-19 suspected). The daily average volume of calls increased from 250 to 350 at the beginning of March to an average of 550 during the last 2 weeks of that month. Most of the increased volume were inappropriate, low acuity requests for information on COVID-19. As we started to document the missions suspected to be related to COVID-19 from March 9th, we noticed the number of COVID-19 mission increased while the volume of non-COVID-19 mission decreased, and therefore the total increase was moderate (10–20%) (Fig. 1). Inappropriate calls were reduced to between five and ten per day once State and nationwide dedicated information helplines were set from March 6th to answers general questions about COVID-19; the State’s help line received 2′500 calls per day. It takes some time for government officials to activate those lines, however, a duration that always seem too long for dispatch centres. These helplines are essential tools to relieve the usual emergency lines, as those calls are not only inappropriate but also last much longer than normal emergency calls. At the same time, the non-emergency help line increased its incoming calls volume from 800 to 2′500 per day.

The second risk that occurs specifically during an event like a pandemic is to see the highly-qualified dispatch personnel fall ill. Dispatchers, unlike doctors and nurses in hospitals, cannot be replaced on short notice. It was therefore initially decided to “sanctify” the dispatch and strictly limit its access to dispatchers and supervisors only. Remote dispatch desks in private homes have been set up and in order to allow regulators suspected or confirmed of COVID-19 infection but still in good health to be able to continue working on a voluntary basis. As a consequence of the large number of dispatchers living on the French side of Lake Geneva (opposite Lausanne), dispatch desks have also been set in operation in France in order to anticipate a possible total closure of the borders. To the best of our knowledge, moving such a sensitive activity to a foreign country is novel. To date, however, no dispatcher has been sick or been confirmed to be positive for COVID-19 infection; the deported desks are still on stand-by, and the border with France is still opened for essential healthcare professionals living in France and working in Switzerland thanks to the good political relations between the two countries.

The third risk dispatch may face during a major incident is the shortage of transportation vectors at their disposal, such as ambulances, either because there is an increased demand or a shortage of resources as paramedics may also be victims of COVID-19. The use of seated transport for low acuity cases that require no care or surveillance offers an increased availability of ambulances for the more serious cases; this has been long-requested in our system and finally granted.

Some additional pending measures include disconnecting the local community’s first-responder system that allows citizen to be alarmed in case of suspected cardiac arrest. Because there are potential risks of contamination for those responders that do not have special equipment and the possible reduced capacity of hospital to take care of cardiac arrests cases, this application may be disconnected at some point. A second measure includes permitting the dispatch’s medical director to downgrade the level of care (outside of protocol) from case to case when resources may be lacking including suspending telephone-CPR, downgrading some missions from ALS to BLS crews, refusing resources for inter-hospital transfers, or to take responsibility for not dispatching an emergency vehicle or allowing a patient to remain at home per the paramedics’ judgment once they are on site.

Some lessons learned so far from COVID-19 include: a non-emergency medical dispatch and specific help lines may save your dispatch from drowning; protect your staff; have a B plan (such as deported-based dispatch); and maintain protocols for downgrading your response. This crisis is likely just beginning. As always, such event is also an opportunity for development and improvement of ongoing procedures.

## Data Availability

Available upon reasonable request.

## Abbreviation

COVID-19: Coronavirus disease 2019
